# Implicit and Explicit Representations of Hand Position in Tool Use

**DOI:** 10.1371/journal.pone.0068471

**Published:** 2013-07-19

**Authors:** Miya K. Rand, Herbert Heuer

**Affiliations:** ***IfADo***-Leibniz Research Centre for Working Environment and Human Factors, Dortmund, Germany; Kyushu University, Japan

## Abstract

Understanding the interactions of visual and proprioceptive information in tool use is important as it is the basis for learning of the tool's kinematic transformation and thus skilled performance. This study investigated how the CNS combines seen cursor positions and felt hand positions under a visuo-motor rotation paradigm. Young and older adult participants performed aiming movements on a digitizer while looking at rotated visual feedback on a monitor. After each movement, they judged either the proprioceptively sensed hand direction or the visually sensed cursor direction. We identified asymmetric mutual biases with a strong visual dominance. Furthermore, we found a number of differences between explicit and implicit judgments of hand directions. The explicit judgments had considerably larger variability than the implicit judgments. The bias toward the cursor direction for the explicit judgments was about twice as strong as for the implicit judgments. The individual biases of explicit and implicit judgments were uncorrelated. Biases of these judgments exhibited opposite sequential effects. Moreover, age-related changes were also different between these judgments. The judgment variability was decreased and the bias toward the cursor direction was increased with increasing age only for the explicit judgments. These results indicate distinct explicit and implicit neural representations of hand direction, similar to the notion of distinct visual systems.

## Introduction

Without a tool, the position of the hand is monitored both visually and proprioceptively, and both modalities are integrated to obtain a single estimate of hand position [1]. Recent evidence shows that the weights in averaging different sources of information match their relative inverse variances [2]–[3]. Such weighted averages are optimal in that they minimize the variance of the combined estimate, and they are appropriate when the information refers to a certain characteristic of one and the same object.

**Figure 1:**
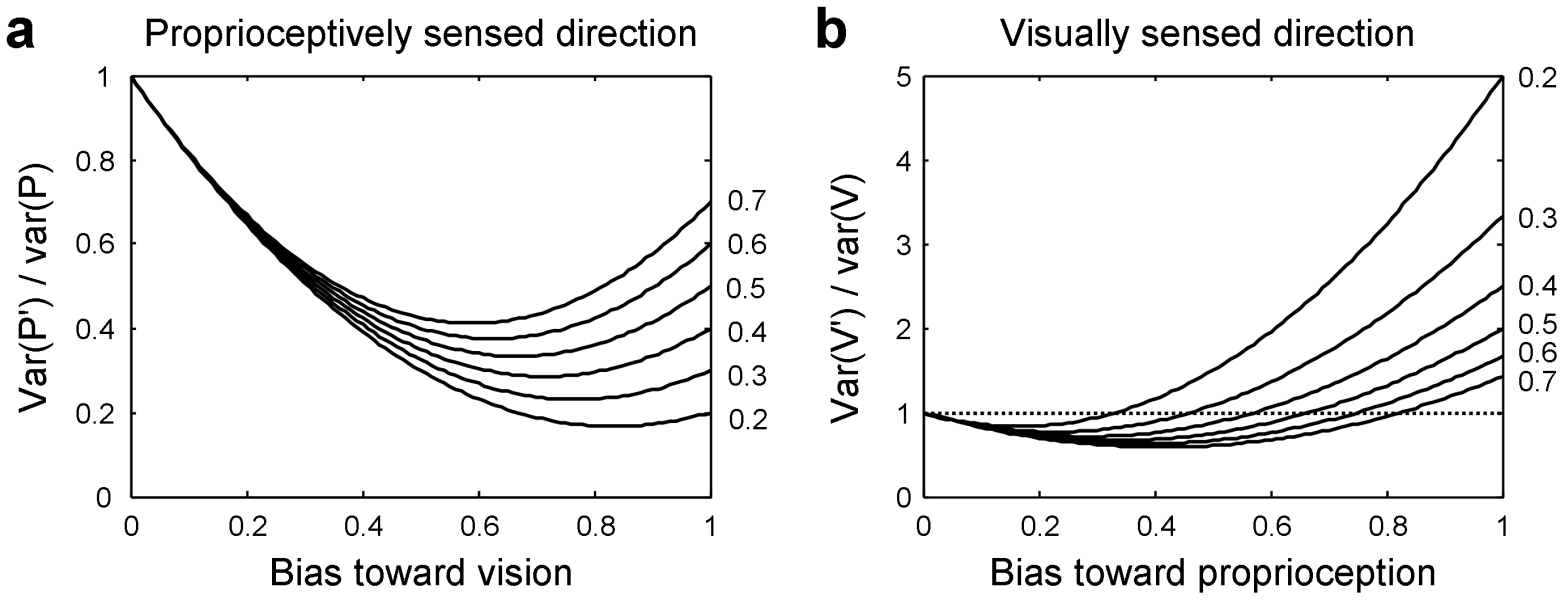
Variance reduction through sensory coupling. Relative variances of biased proprioceptively (a: var(*P*')/var(*P*)) and visually (b: var(*V*')/var(*V*)) sensed spatial characteristics, such as directions, are plotted against the proportional bias toward the other modality, vision and proprioception, respectively. Var(*P*') and var(*V*') are the variances of the biased directions, whereas var(*P*) and var(*V*) are the variances of the unbiased directions based only on proprioception and vision, respectively. The relative variances are plotted as a function of bias for different ratios of var(*V*)/var(*P*) (0.2 to 0.7). Equations are given in Appendix (Text S1). Note that sensory coupling serves to reduce variability (a, b) to a minimum at an intermediate bias, and a weak coupling (small bias) does so even for the more precise visually sensed spatial characteristic (b).

In tool-use actions, such as controlling a cursor on a monitor through a computer mouse, however, visual information specifies the position of the effective part of the tool (i.e., cursor), while proprioceptive information specifies the position of the hand. These two positions of different objects have a clear spatial separation. They are related to each other only through the tool's kinematic transformation. Even though the positions of the hand and the cursor remain distinct perceptually, they might be biased toward each other. This kind of interaction between sensory signals, which does not result in a fused percept but in distinct perceptions with mutual biases, has been referred to as coupling [4]. Coupling can also be conceived in terms of weighted averages of the different sensory signals (see Appendix – Text S1). It can serve to reduce the variances of the biased estimates, and thus, to enhance the precision even when visual and proprioceptive information refer to different objects. This is illustrated in Figure 1. Note that even the smaller variance of the visually based perceptions can be reduced by sensory coupling.

The coupling of visual and proprioceptive spatial information in tool-use actions is not well understood. Thus, this study investigated sensory coupling and the resulting perceptual biases in tool use. Proprioceptive biases toward vision have been observed previously (i.e., visual capture [5]), whereas visual biases toward proprioception might be of little importance in tool-use actions, as attention tends to be focused on the visual information [6]–[7] and conscious awareness of the position of the hand becomes severely limited [8]. Indeed, there is both behavioral [9] and electrophysiological [10] evidence of functional neglect and suppression of proprioceptive input to the somatosensory cortex during tool-use actions. The absence or functional impairment of proprioceptive information can be even beneficial for tool-use performance [11]–[12]. Based on these considerations, we tested the hypothesis that the proprioceptively sensed direction of the hand is strongly biased toward the visually sensed direction of the cursor in tool use (as visual capture [5]), whereas the latter is only slightly biased toward the former.

Coupling of the proprioceptive information on hand direction and the visual information on cursor direction together with the resulting perceptual biases should be associated with costs in terms of one's overall learning capability of the tool's kinematic transformation. In particular, it should reduce the explicitly known differences between hand positions, that constitute the input to the transformation, and cursor positions, that constitute the output. Thereby it should compromise the explicit learning of the relation between hand and cursor positions, which is an element of learning of kinematic transformations [13].

Learning of a tool's transformation, however, also involves an implicit process that is outside conscious awareness [14]–[15]. This process requires hand-position information as well, although its sources might differ from the sources of conscious awareness of hand position. For example, motor outflow or the associated corollary discharge [16] or efference copy [17] might be involved to a greater extent. Accordingly, it should be possible to identify a second, implicit rather than explicit, representation of the direction of the hand. For this purpose, we devised a task that enables an indirect (or implicit) measure of the sensed hand direction [18]. This measure exploits error propagation in successive aiming movements [19]–[21], in particular the propagation of errors that originate from visually induced discrepancies between the physical and the perceived position of the hand [22]–[24].

In the present study, the relation between the explicit and implicit measures of hand direction was explored in three different ways. First, we determined the inter-individual covariation of both measures, which should be close to zero if they indeed tap distinct representations. Second, we tested age-related variations for the two measures, which could be different. Assume that the explicit measure taps a representation being used for explicit learning of kinematic transformations and that the implicit measure taps a representation being used for implicit learning. Further assume that learning suffers when perceptual biases become stronger, so that the directions of hand and cursor become harder to discriminate. Then, the observation of age-related deficits of explicit learning [13], [25] leads one to expect stronger perceptual biases in older than in younger adults for the explicit measures, in particular for the stronger bias of the explicit measure of the sensed direction of the hand toward the direction of the cursor. In contrast, there should be no age-related variation for the implicit measure because there is no age-related deficit of implicit learning [13], [25]. Third, we tested sequential effects of trials for explicit and implicit measures which could be different as well. In the present study, one of the two types (cursor and hand) of explicit judgment was randomly instructed to the participants for each trial, thus possibly enabling for participants to relate to trial history regarding the type of judgment. Hence, it could affect information processing in the subsequent trials.

The results will show mutual biases with a strong asymmetry, namely, a visual dominance over proprioception. Importantly, implicit and explicit judgments of hand direction exhibited a number of differences, among them different age-related changes, that strongly suggest that they tap different neural representations.

## Materials and Methods

### Participants

Twenty young adults (mean±SD: 26.1±3.2 years; range: 20–30 years; 9 males and 11 females) and twenty older adults (59.6±5.7 years; range: 50–69 years; 10 males and 10 females) participated in the study. All participants were right-handed, had no history of stroke, arthritis, or other neurological or movement impairments, and gave written informed consent prior to participation. The study was conducted in accordance with the Declaration of Helsinki and with general approval by the ethics committee of the Leibniz Research Centre for Working Environment and Human Factors.

Young and older participants were compared on two subtests of the German version of the Wechsler Adult Intelligence Scale [26]: the Digit Symbol Test, a test of perceptuo-motor processing speed, and the Vocabulary Test, a test of culturally mediated knowledge. Consistent with typical findings, the average score on the Digit Symbol Test was significantly higher for the young adults (mean±SD: 66.7±12.1) than for the older adults (50.9±13.6, *t*(38) = 3.8, *P*<0.001), whereas the average score of the Vocabulary Test tended to be lower for the young adults (25.8±3.9) than for the older adults (30.6±11.3, *t*(38) = 1.7, *P* = 0.088). According to these findings, the two groups were representative for their respective age groups in terms of general age-related variations and invariances.

### Apparatus

**Figure 2:**
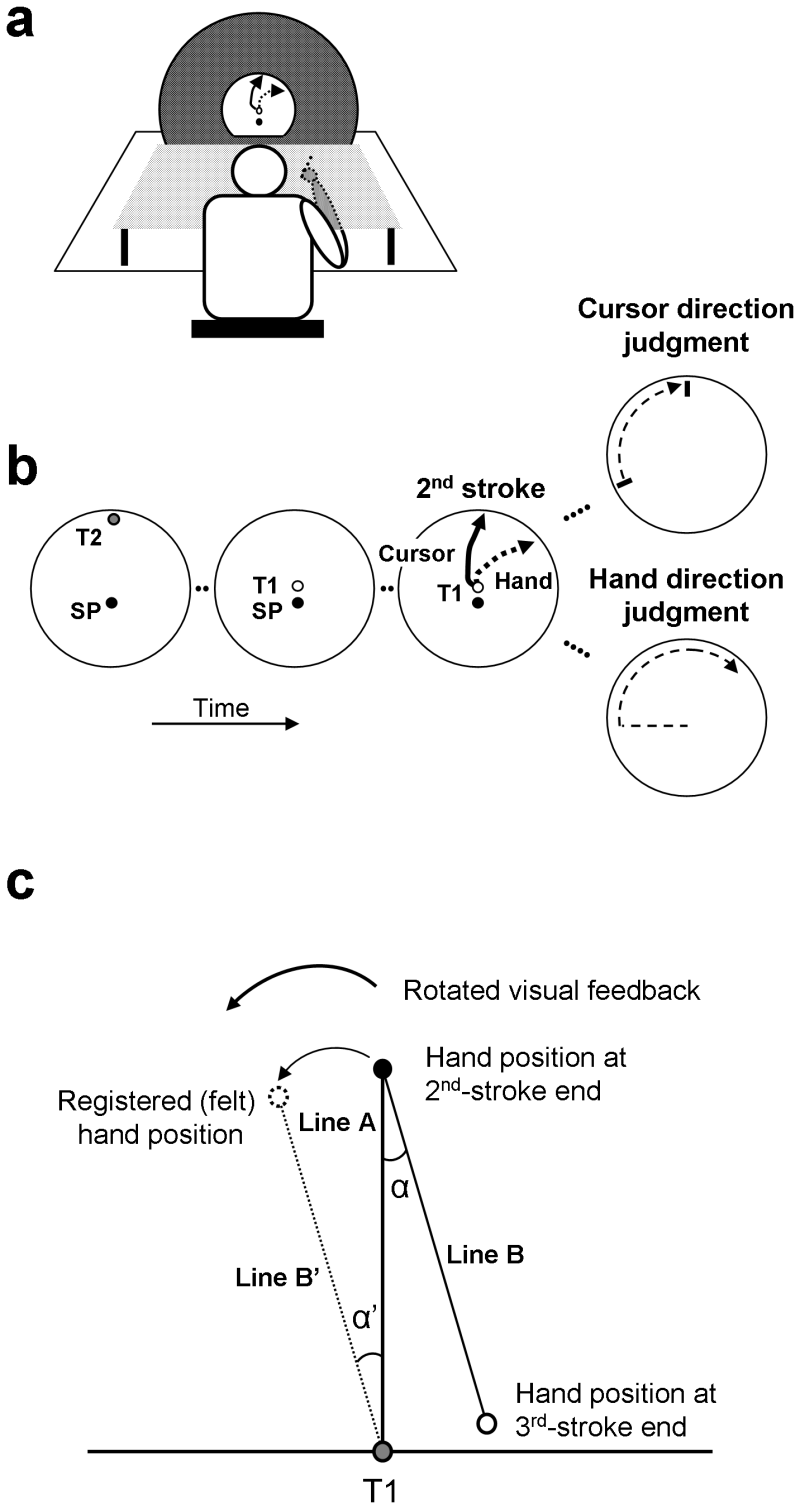
Behavioral task of a 3-stroke movement and analysis. (a) The experimental setup. (b) The judgment task of the hand and cursor conditions. SP, T1 and T2 refer to a starting position, a first target, and a second target, respectively. The visual feedback of the 2^nd^-stroke is rotated and displayed simultaneously with hand movements. After each movement, the participants make an explicit judgment regarding the hand or cursor direction at the end of the 2nd-stroke. (c) The illustration of the relationship between the actual hand position (black circle) at the end of the 2nd-stroke and its felt hand position (dotted outline circle), which is estimated based on the shift of the hand position at the end of the 3rd-stroke (solid outline circle) from the first target (T1, grey circle). Angle α' is calculated as the angular deviation of the implicit judgment of hand direction.

The experimental setup (Fig. 2a) was quite similar to the one used in our previous study [18]. Participants were seated at a table, on which a digitizer tablet (133 Hz sampling rate) and a monitor were placed. The monitor was covered by a large black circular screen with a semi-circular window (32 cm in diameter) in its center. The participants held a stylus with their right hand and made movements on the digitizer. An opaque board placed above the participants' arm blocked their direct view of the hand movements.

### Design and procedure

Participants performed three-stroke arm movements as described previously [18]. The first target (T1, 1.4 cm in diameter) was located in the center of the semi-circular window (Fig. 2b). The start position (SP, 1.2 cm in diameter) was located 3 cm below T1. A second target (T2, 1 cm in diameter) was presented at pseudo random locations, ranging from −60° to +60° relative to the central location, on an invisible circle with a radius of 15 cm around T1. The participants made three-stroke movements from the SP to T1 (1^st^ stroke), then to T2 (2^nd^ stroke), and subsequently back to T1 (3^rd^ stroke). To stop the movements mechanically at the end of the 2^nd^ strokes, a semi-circular plastic ring (stopper ring) with a radius of 15 cm around T1 and 3 mm height was placed on the digitizer's surface.

At the beginning of each trial, participants were guided to the SP by arrows shown on the monitor [18]. One second after the stylus was in the SP, T2 was displayed for 1 s (Fig. 2b, 1^st^ panel). Subsequently, T1 was displayed. After a delay of 0.5 s, an auditory go-signal was delivered. The participants then made three-stroke movements at a comfortable speed. Once the participants made the 1^st^ stroke to T1, this target disappeared. The 1^st^ stroke was introduced because the participants would naturally look at T1 [27]–[28], which prevented them from keeping their gaze on T2 to remember its location. Subsequently, the participants made the 2^nd^ stroke to the remembered T2 until the movement was stopped by the stopper ring, and then made a return movement (3^rd^ stroke) back to the remembered T1 location.

The feedback cursor was displayed concurrently with the hand movements only during the 1^st^ and the 2^nd^ strokes. Only during the 2^nd^ strokes, the motions of the cursor were rotated relative to the directions of the hand movements. Participants had to adjust their movements so that the cursor on the monitor would move toward the remembered T2 location (Fig. 2b, 3^rd^ panel). The remembered T2 was introduced instead of a visible T2, so that the participants focused on the visual feedback cursor rather than on the visual target during the 2^nd^ stroke. There were twelve different rotation angles (clockwise [CW] direction: −30°, −25°, −20°, −15°, −10°, −5°; counter-clockwise [CCW] direction: 5°, 10°, 15°, 20°, 25°, 30°), which were randomized across trials.

The participants made the 3^rd^ stroke without visual feedback. One second after completing the 3^rd^ stroke, the participants were asked to judge either the hand or cursor direction at the end of the 2^nd^ stroke (Fig. 2b, 4^th^ panels). For the judgment of cursor direction (explicit cursor judgment, 4^th^ panel top), a short white line (width: 0.15 cm; length: 2 cm) was displayed. It marked the peripheral end of a radial line from T1 to the circumference of the invisible ring of 15 cm diameter centered at T1. The radial line, and thus its visible peripheral end, moved at a constant speed counter-clockwise or clockwise, beginning at a start position 102° to the right or left of the vertical. The participants instructed the examiner to stop and finely adjust the line to the direction that matched the direction of the cursor at the end of the 2^nd^ stroke. For the judgment of hand direction (explicit hand judgment, 4^th^ panel bottom), the participants moved the pen from the right (or left) lower corner of the stopper ring counter-clockwise (or clockwise) along the ring and stopped where he/she thought the hand direction matched the direction of the hand at the end of the 2^nd^ stroke. This hand movement along the circular path served to indicate the hand position at the end of the 2^nd^ stroke instead of reproducing the movement from motor memory. For the explicit judgments, type of judgment (hand or cursor direction) and direction of line or hand movement during the judgment (clockwise or counter-clockwise) were randomized across trials.

Data were recorded in six trials for each of the 12 angular rotations of visual feedback during the 2^nd^ stroke, totaling 72 trials for each type of explicit judgment, cursor and hand. The experiment consisted of a block of 5 familiarization trials, a block of 8 practice trials, and a block of experimental trials (144 experimental trials were preceded by a warm-up trial). The familiarization trials included the procedure without the visual-feedback rotation and without the judgment part. The practice trials included all the procedure. A couple of breaks of a few minutes each were inserted as needed during the block of experimental trials.

### Data Analysis

#### Explicit judgments of cursor and hand direction

The angular deviation of the judged hand (or cursor) direction from the actual hand (or cursor) direction at the end of the 2^nd^ stroke was measured in each trial. A positive (or negative) angular deviation indicated a CCW (or CW) deviation of the judged direction from the actual direction.

Means and standard deviations of the angular deviations were computed for each type of judgment (cursor or hand) and each visual-feedback rotation in each participant. To measure the influence of the rotated visual feedback on the explicit judgments, a linear regression was performed for each participant and each type of judgment; angular deviation was set as the dependent variable and visual-feedback rotation as the independent variable. The resulting slope parameters are estimates of the proportional biases of the explicit judgments. They specify the strength of the coupling in terms of the biases of both types of judgment in degrees per degree of the visual-feedback rotation. We shall refer to them as explicit-judgment biases. The intercepts of the regression lines served to assess overall offsets of the judgments in the CCW or CW direction independent of the visual-feedback rotation.

The data were screened for outliers both among trials and among participants. Based on the linear regressions, trials with judgments outside the range of predicted judgments ±3SD of the residuals were eliminated as outliers. As a result, 28 trials (0.49 %) out of 5,760 trials were removed from all analyses. Subsequently, the bias parameters for each type of judgment and each participant were screened for outliers. Means and standard deviations across all participants of both age groups were calculated for the two types of judgment, and bias parameters outside the range of mean±3SD were defined as outliers. These computations were repeated until no further outliers were found. As the result, two young adults and one older adult were identified as having outliers for the cursor judgments, and another young adult for the hand judgments. These participants were excluded from all analyses.

#### Indirect measure of the sensed direction of the hand

The indirect (implicit) measure of the sensed direction of the hand was computed as described previously [18]. The following three steps were taken. First, the angle (α in Fig. 2c) between the line connecting T1 with the end of the 2^nd^ stroke (Line A) and the line connecting the end of the 2^nd^ stroke with the end of the 3^rd^ stroke (Line B) was measured. Second, Line B was shifted in parallel to the axes of the coordinate system until its end (i.e., the end of the 3^rd^-stroke, white open circle in Fig. 2c) was in T1. Third, the location of the other end of the shifted line (Line B') served as an estimate of the felt hand position at the end of the 2^nd^-stroke (dotted white circle in Fig. 2c). We used the angle α' between the Line A and the Line B' as estimate of the rotation of the felt hand position relative to the actual hand position, that is, as implicit angular deviation. When Line B' (i.e., 3^rd^ stroke) was rotated to the CCW or CW direction compared to Line A (i.e., 2^nd^ stroke), the implicit angular deviation (α') had a positive or negative sign, respectively.

The implicit judgment was made in each trial before the type of explicit judgment was instructed to the participants. Therefore, means and standard deviations of the implicit angular deviations were computed across both types of explicit judgment for each visual-feedback rotation and each participant. To measure the influence of the rotated visual feedback on the indirect measure of the sensed hand direction, the implicit angular deviations (α') of each participant were subjected to a linear regression with rotation of visual feedback as the independent variable. The resulting slope parameter is an estimate of the bias of the indirect measure of the sensed hand direction per degree of the rotation of visual feedback. We shall refer to it as implicit-judgment bias. The intercept of the regression line is an estimate of the overall offset of the implicit judgments in the CCW or CW direction independent of the visual-feedback rotation.

Trials were screened for those with outlying implicit measures by using the same principles as described above for the explicit judgments. Based on the linear regressions, 19 trials (0.33%) out of 5,760 trials were removed from all analyses. Next, the individual bias parameters were screened for outliers; another young adult was excluded from all analyses, leaving 16 young adults and 19 older adults for the subsequent analyses.

#### Statistical analyses

Individual means, standard deviations, and regression coefficients as well as intercepts were subjected to statistical analyses. These were mainly ANOVAs with the between-participant factor age group (young vs older) and different within-participant factors, such as rotation of visual feedback or type of judgment. The specific within-participant factor(s) used are stated in the Results section. When appropriate, post-hoc comparisons were performed using t-tests with Bonferroni correction (α = 0.05). Additionally, the individually computed slopes and intercepts of the linear regressions were subjected to one-sample t-tests against zero. To examine whether the implicit-judgment bias of hand direction was related to the explicit-judgment bias of hand direction and/or cursor direction, the correlations between them were calculated for both age groups.

For the analysis of sequential effects of the type of explicit judgment on implicit- and explicit-judgment biases, trials were classified into four categories according to two characteristics of the preceding trials: 1) the preceding trial required an explicit hand judgment or a cursor judgment; 2) the type of explicit judgment in the preceding trial was a repetition or not. Explicit-judgment biases of hand and cursor direction and implicit-judgment bias of hand direction were computed for each participant and each subset of trials.

## Results

### Explicit judgments of cursor and hand directions

**Figure 3:**
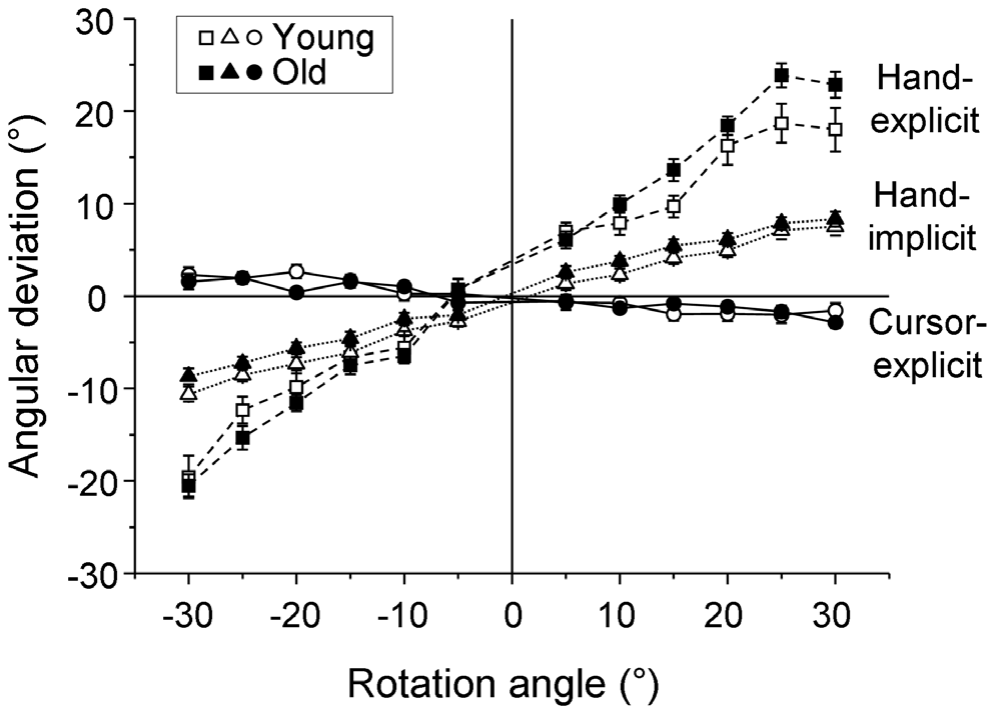
Implicit and explicit judgments. Mean angular deviations of the judged directions from the corresponding physical directions as a function of the rotation of visual feedback. The values are plotted for explicit judgments of cursor direction (circles) and hand direction (squares) and implicit judgments of hand direction (triangles). White and black symbols refer to the young and older groups, respectively. The error bars represent the SE.

The mean angular deviations of the judged cursor directions from the corresponding physical directions showed a slightly negative slope as a function of the visual-feedback rotation (Fig. 3, circles). This indicates a slight bias of the cursor judgments toward the direction of the hand. Conversely, the mean angular deviations of the judged hand directions from the physical directions showed a steep positive slope as a function of the feedback rotation (Fig. 3, squares). This indicates a strong bias of the hand judgments toward the direction of the cursor. The older adults had a greater slope than the young adults, hence their bias was stronger.

Bias parameters were computed individually as slopes of the individual regressions of angular deviations on visual-feedback rotations. The mean (±SE) biases of explicit cursor judgments were slightly negative for both the young (−0.08±0.02) and older (−0.07±0.01) groups. One-sample t-tests revealed that the means were significantly below 0 for both groups (young: *t*(15) = −4.4, p<0.001; older: *t*(18) = −5.7, p<0.001). Conversely, the mean (±SE) biases of explicit hand judgments were strongly positive (young: 0.63±0.04; older: 0.75±0.03). A 2 (group: young vs older) x 2 (type of judgment: cursor vs hand) ANOVA showed that the main effects of group (*F*(1,33) = 5.5, *P*<0.05) and type of judgment (*F*(1,33) = 910.7, *P*<0.001) were significant, and so was the group by type-of-judgment interaction (*F*(1,33) = 4.4, *P*<0.05). The interaction effect was due to a significantly stronger bias of the explicit hand judgments for the older adults compared to the young ones (*P*<0.05), while there was no group difference for the bias of the explicit cursor judgments (*P*>0.05).

### Implicit and explicit judgments of hand direction

Similar to the explicit judgments, mean angular deviations of the implicit hand judgments had a positive slope as a function of the visual-feedback rotation (Fig. 3, triangles), indicating a bias toward the cursor direction. The mean (±SE) biases of implicit hand judgments were 0.31±0.02 for the young and 0.30±0.02 for the older participants. These means were not different between the two age groups, and they were smaller than the explicit-judgment biases. A 2 (group: young vs older) ×2 (type of judgment: hand-implicit vs hand-explicit) ANOVA revealed a significant main effect of type of judgment (*F*(1,33) = 219.3, *P*<0.001) and a significant interaction (*F*(1,33) = 6.6, *P*<0.05). The interaction effect was due to the significantly stronger bias of the explicit hand judgments in the older group than in the young group, while there was no group difference for the bias of the implicit hand judgments (*P*>0.05).

**Figure 4:**
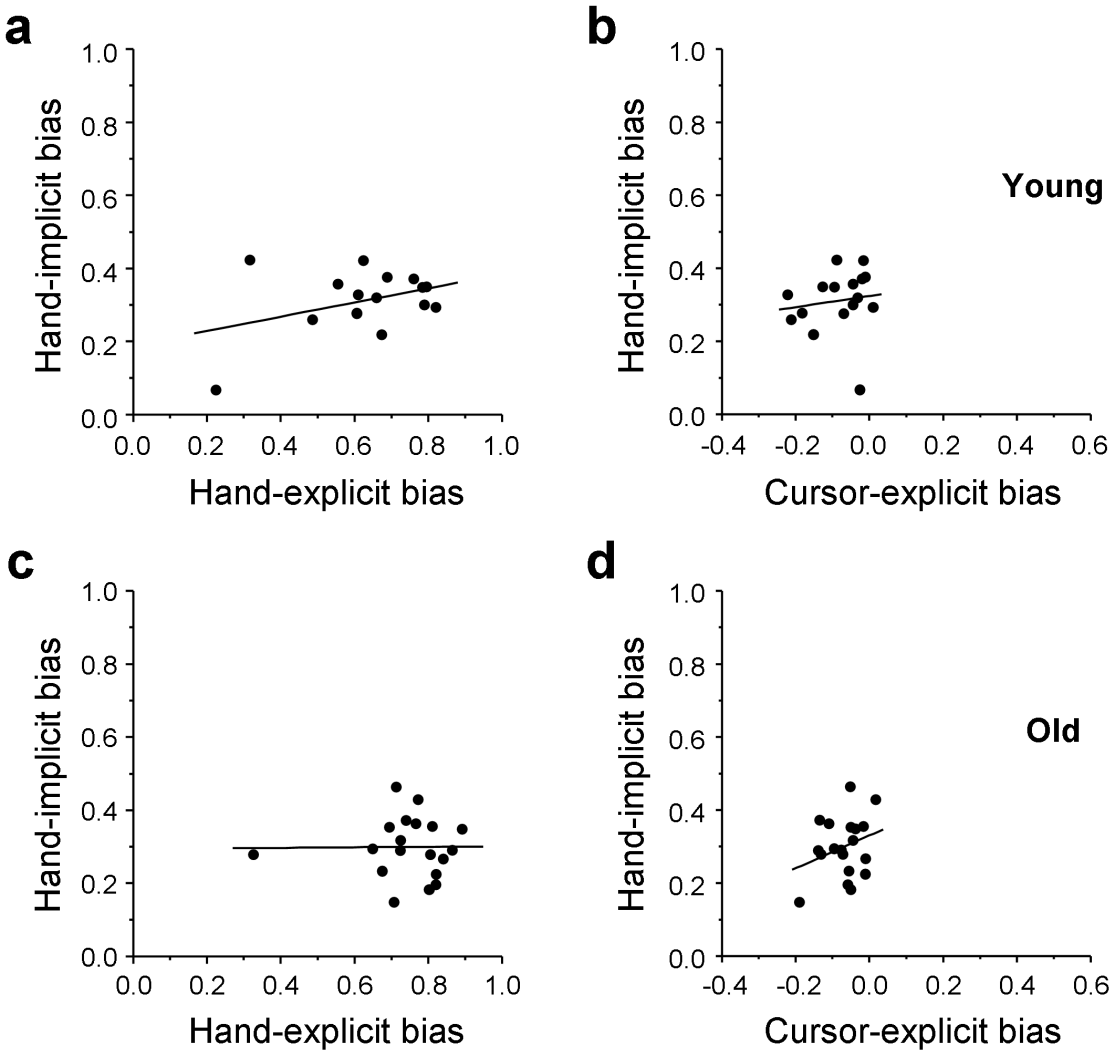
Correlations between explicit- and implicit-judgment biases. Scatter plots of the relations between the individual implicit-judgment biases of hand direction and the individual explicit-judgment biases of hand direction (a, c) and cursor direction (b, d) in the young (a, b) and older (c, d) group.

The presence of an age effect on the explicit-judgment bias, but not on the implicit-judgment bias, supports the hypothesis that the different measures tap different representations of arm direction and are not derived from a single representation. As a further test of this hypothesis, we computed the correlations between the two types of bias in the two age groups (Fig. 4a, c). Neither for the young (Fig. 4a, *r* = 0.381, *P*>0.05) nor for the older participants (Fig. 4c, *r* = 0.009, *P*>0.05) was the correlation statistically significant. In addition, we also tested whether the implicit-judgment bias of hand direction was related to the explicit-judgment bias of cursor direction (Fig. 4b, d). In both age groups, the correlations were not significant (young: *r* = 0.133, *P>*0.05, Fig. 4b; older: *r* = 0.288, *P>*0.05, Fig. 4d), showing that these two types of biases were also not related to each other.

### Offsets of implicit and explicit judgments

The mean angular deviation of explicit hand judgments was a few degrees above zero at the 0° rotation of visual feedback for both age groups (Fig. 3, squares). This indicates that participants generally judged the direction of the hand to be slightly rotated in the counter-clockwise direction compared to the physical direction. In contrast, the mean angular deviations of explicit cursor judgments (Fig. 3, circles) and of implicit hand judgments (Fig. 3, triangles) were around zero for both age groups, indicating that there was no such offset of judged directions relative to physical ones. One-sample t-tests were applied to the intercepts of the individual regressions of angular deviations on visual-feedback rotations. The mean intercept of the explicit hand judgments was significantly greater than zero (*t*(34) = 3.4, p<0.001), whereas the mean intercepts of the other two types of judgment did not differ from zero (*P*>0.05).

### Intra-individual variability of judgments

**Figure 5:**
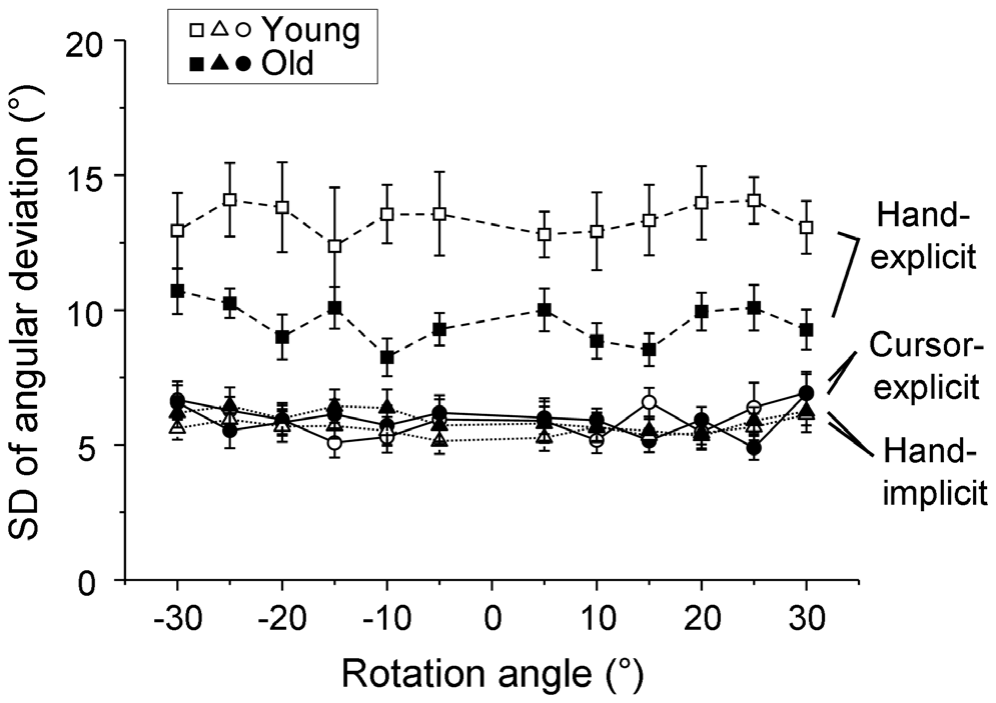
Intra-individual variability of judgments. Mean standard deviations (SD) of the angular deviations of the judged directions from the corresponding physical directions as a function of the rotation of visual feedback. The values are plotted for explicit judgments of cursor direction (circles) and hand direction (squares) and implicit judgments of hand direction (triangles) Open and filled symbols refer to the young and older groups, respectively. The error bars represent the SE.

The asymmetric biases of visually and proprioceptively sensed directions are likely to be related to their respective precisions [29]–[30]. Therefore, we measured the variability of explicit and implicit judgments. The mean intra-individual standard deviations were small and similar for the explicit cursor judgments (Fig. 5, circles) and the implicit hand judgments (Fig. 5, triangles), but much larger for the explicit hand judgments (Fig. 5, squares). Only for the latter type of judgment, the young participants had a greater intra-individual variability than the older participants.

The individual standard deviations were subjected to a 2 (group: young vs older) ×3 (type of judgment: cursor-explicit, hand-explicit, and hand-implicit) ×12 (visual-feedback rotation) ANOVA. The interaction of group and type of judgment was significant (*F*(2,66) = 9.1, *P*<0.001), and so were the main effects of group (*F*(1,33) = 7.5, *P*<0.001) and type of judgment (*F*(2,66) = 71.2, *P*<0.001). The explicit hand judgments showed significantly greater variability than the other two types of judgment (post-hoc, *P*<0.001), which were not significantly different from each other (*P*>0.05). Furthermore, the observed interaction effect was due to a significant group difference for the explicit hand judgments (*P*<0.001), but not for the other two types of judgment (*P*>0.05). There was no significant effect of visual-feedback rotation.

### Sequential effects of the type of explicit judgment

The type of explicit judgment was instructed to the participants only after the three-stroke movement had been finished in the present experiment. Thus, this instruction should not affect the processing of the visual and proprioceptive information on cursor direction and hand direction in the current trial. However, in principle it could affect information processing in the subsequent trial. Therefore, we examined the sequential effects of the type of explicit judgment on the biases both of explicit and implicit judgments.

**Figure 6:**
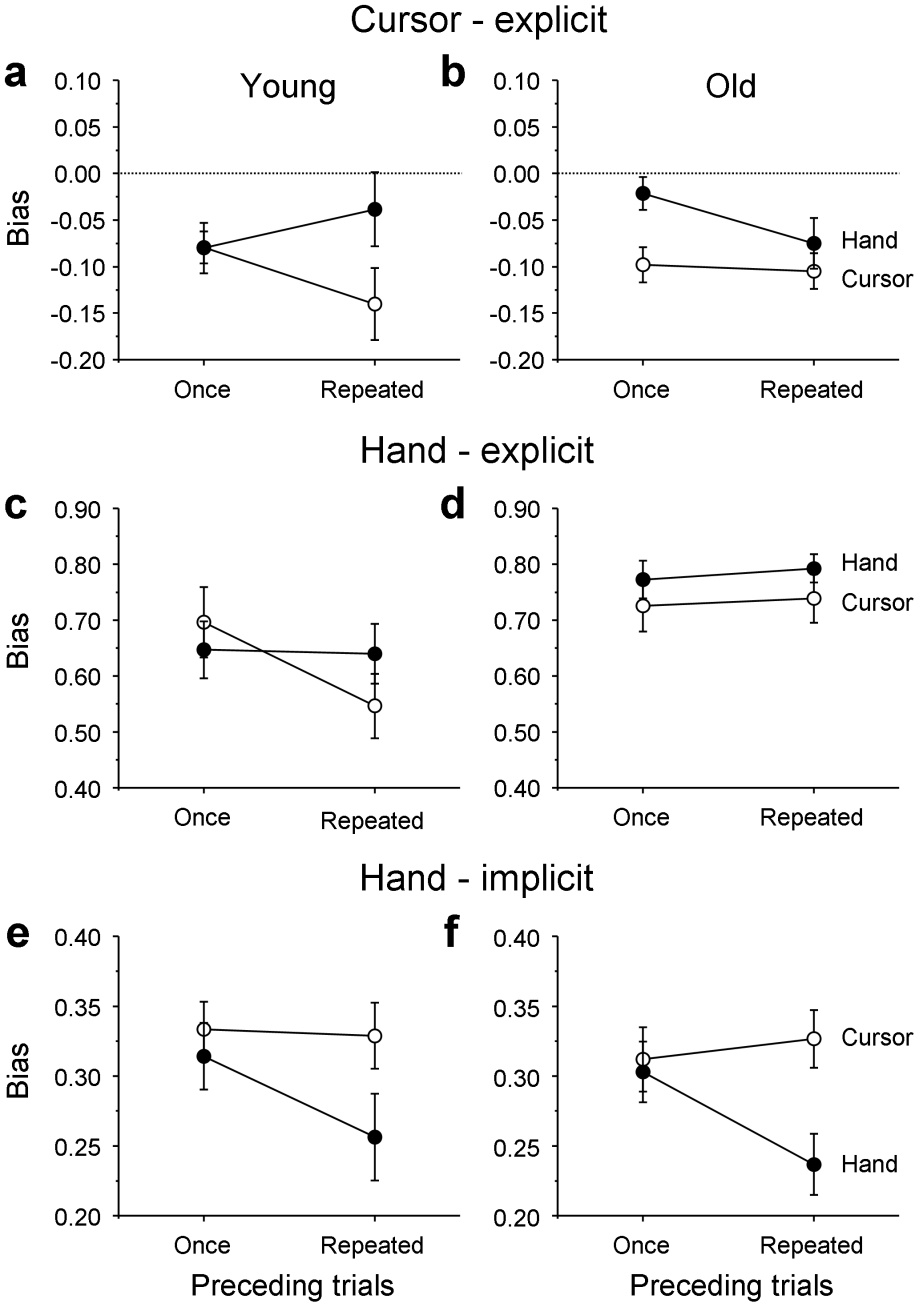
Sequential effects on judgment biases. Sequential effects of the type of explicit judgment on the explicit-judgment biases of cursor direction (a, b) as well as explicit- (c, d) and implicit-judgment biases of hand direction (e, f) in the young (a, c, e) and older (b, d, f) age groups. The mean biases are plotted separately depending on the type (cursor direction: open circles; hand direction: filled circles) of the explicit judgment performed in the preceding trials and on its repetition (once or repeated). The error bars represent the SE.

The bias of the explicit hand judgments was somewhat stronger (greater positive values) after explicit hand judgments (Fig. 6c, d, filled circles) than after explicit cursor judgments (open circles). Similarly, the bias of the explicit cursor judgments was generally stronger (smaller negative values) after explicit cursor judgments (Fig. 6a, b, open circles) than after explicit hand judgments (filled circles). These results indicate that the biases of explicit judgments became stronger when the same type of explicit judgment was required in preceding trials. In terms of coupling, this implies that the weight of that sensory information was increased that was not relevant for the preceding explicit judgments. Such effects were consistently found in the older adults regardless of the number of judgment repetitions (Fig. 6b, d), but observed only after repeated judgments in the young adults (Fig. 6a, c).

The means of the individually determined biases of explicit judgments were subjected to a 2 (group: young vs older) ×2 (type of judgment: cursor vs hand) ×2 (preceding type of explicit judgment: cursor vs hand) ×2 (repetition: once vs repeated) ANOVA. For this analysis, the sign of all bias values of the explicit cursor judgments (Fig. 6a, b) was changed to the opposite so that larger values indicated stronger biases as was the case with the explicit hand judgments (Fig. 6c, d). The two-way interaction of type of judgment and preceding type of judgment was significant (*F*(1,33) = 7.9, *P*<0.01), confirming the above observation that biases of explicit judgments became relatively stronger when the explicit judgments were repeated. Additionally, there were significant main effects of group (*F*(1,33) = 5.2, *P*<0.05) and type of judgment (*F*(1,33) = 450.2, *P*<0.001), as well as significant interactions of type of judgment and group (*F*(1,33) = 5.3, *P*<0.05), repetition and group (*F*(1,33) = 4.7, *P*<0.05), and of all four factors (*F*(1,33) = 5.0, *P*<0.05).

The mean bias of implicit hand judgments (Figure 6e, f) was generally weaker after explicit hand judgments (filled circles) than after explicit cursor judgments (open circles) in both age groups. This difference was much emphasized after repeated explicit judgments of hand direction. In terms of coupling, it implies a stronger weight of hand-position information after explicit judgments of hand position rather than a stronger weight of cursor-position information. These findings contrast sharply with the sequential effects found for the explicit hand judgments. We performed a 2 (group: young vs older) ×2 (type of judgment: hand-explicit vs hand-implicit) ×2 (preceding type of explicit judgment: cursor vs hand) ×2 (repetition: once vs repeated) ANOVA. A significant two-way interaction of type of judgment and preceding type of judgment (*F*(1,33) = 7.7, *P*<0.01) and a significant three-way interaction of type of judgment, preceding type of judgment, and repetition (*F*(1,33) = 6.6, *P*<0.05) confirmed the above observations. Other significant main effects or interactions were type of judgment (*F*(1,33) = 238.7, *P*<0.001), repetition (*F*(1,33) = 6.1, *P*<0.05), type-of-judgment by group (*F*(1,33) = 7.3, *P*<0.05), and repetition by group (*F*(1,33) = 4.4, *P*<0.05).

In summary, biases of explicit judgments tended to become stronger when the same type of explicit judgment was required in preceding trials, in particular after more than one trial with that type of judgment. In contrast, the bias of implicit hand judgments became weaker after explicit hand judgments in the preceding trials. Moreover, the sequential effects of the type of explicit judgment in the preceding trials implicit-judgment biases in the current trials were similar across the two age groups, but tended to be different for the explicit-judgment biases.

## Discussion

### Asymmetric biases of cursor judgments and hand judgments

The present results revealed strong mutual biases of perceived spatial characteristics of the cursor and the hand, being consistent with previous studies [8], [18], [31]. Instead of full information integration, the perceived directions of hand and cursor remained different. The sum of the proportional biases of cursor and hand judgments was less than 1, but larger than 0. This indicates sensory coupling, a sensory interaction, which is intermediate on the continuum between perfect integration or fusion and independence [32].

The biases showed visual dominance over proprioception. Visual dominance is likely related to the higher precision of visual than of proprioceptive spatial information. For example, visual dominance can be turned into proprioceptive dominance under conditions where visual information has lower precision than proprioceptive information [29]–[30]. Consistent with a critical role of relative precision, we observed a higher variability of explicit judgments of hand direction compared to cursor direction. An alternative account of the visual dominance found in the present study could refer to the nature of the task, the control of a visually perceived cursor, which could go along with an attentional focus on cursor direction rather than on hand direction. However, bimodal integration is likely not affected by modality-specific attention [33].

The present findings suggest the conclusion that, for coupling of the two distinct types of spatial information in tool use, the central nervous system implements basically the same mechanism as in the case of sensory integration without tool use. In fact, coupling in tool use can be conceptualized as a generalization of sensory integration which results in biases rather than in fusion (see Appendix – Text S1). It likely produces more precise estimates of cursor and hand directions, but at the cost of systematic errors that result in inaccurate information on the tool's kinematic transformation.

The bias of the hand-direction judgments towards the cursor direction became stronger at older age, whereas the bias of the cursor-direction judgments towards the hand direction remained unchanged. The stronger bias at older age should reduce the sensitivity to sensory conflicts [18], [31] and impair the learning of the relation between hand positions and cursor positions.

Aging not only results in stronger biases overall but also enhances the asymmetry of the biases. This likely is a result of the increased reliance on vision at older age [34]–[36]. A consequence of this age-related change could be the smaller variability of the explicit hand judgments for the older than for the young adults. This finding is puzzling at first because larger, rather than smaller, variability of the judgments is expected in older adults for two reasons. First, older adults are known to have poorer proprioceptive sensitivity [37]; even though it is not always the case [38], higher sensitivity of older adults would be quite unusual. Second, and more generally, aging increases the neural noise of information processing [39]–[40]. However, the expectation of a larger variability in older adults was not confirmed in this study. The smaller variability found instead is likely a by-product of their stronger bias. As is illustrated in Fig. 1, the variability of coupled sensory signals declines as the coupling and thus the bias becomes stronger until a minimum is reached. In support of such critical role of bias, the age-related decline of variability was found only for the explicit hand judgments where the bias was increased, but not for the explicit cursor judgments and the implicit hand judgments where the biases were invariant across the age range studied.

### The dissociation of explicit and implicit judgments of hand direction

Our findings revealed a number of differences between the explicit and implicit judgments of hand direction. First, explicit judgments had considerably larger variability than implicit judgments. Second, only the explicit judgments exhibited an offset in counter-clockwise direction. Third, the bias toward the direction of the cursor for the explicit judgments was about twice as strong as for the implicit judgments. Fourth, the bias toward the cursor direction increased with age only for the explicit judgments. Fifth, the variability declined with increasing age only for explicit judgments. Sixth, the individual biases of explicit and implicit judgments were uncorrelated. Seventh, biases of explicit and implicit judgments exhibited opposite sequential effects.

Taken together, these findings suggest the existence of two different representations of hand direction, similar to different representations of visual stimuli (e.g., [41]–[42]). At present, the functional distinction of an implicit and an explicit representation of hand direction cannot be mapped to particular regions of the brain. However, different neural substrates, such as the posterior-parietal cortex [43]–[45], the primary motor cortex [46], and the premotor cortex [47] could be involved. Both the implicit and the explicit measures of hand direction are affected by visual direction information according to the present results. Consistent with this observation, at least some of these neural substrates that are involved in processing hand direction combine proprioceptive and visual information. Different representations of hand direction can also be based on different combinations of the available sources of information. For example, the explicit representation could rely more on signals of the different types of sense organs in the joints, ligaments, and muscles, whereas the implicit representation could rely more on motor-outflow related information.

The observed differences between implicit and explicit judgments of hand direction, however, do not necessarily result from different representations. They can also originate from other factors when a single representation of hand direction is accessed by different response systems and/or at different times. Such issues play a central role in controversies around the notions of two visual systems (e.g. [48]–[49]) or of implicit and explicit learning systems (e.g. [50]).

For example, some of the observed differences between implicit and explicit hand judgments could result from the decay characteristics of a single representation of hand direction. Our task included a conspicuous temporal difference between implicit and explicit judgments that might have contributed to their different characteristics: implicit judgments were inferred from the immediate return movements after the end of the second stroke of each trial, whereas explicit judgments were finished about 5.7 s later on average. When a single representation of hand direction decays with the passage of time, its precision declines, and the variability of responses based on it increases, and so do eventual constant errors (e.g. [51]). Thus, the later explicit judgments of hand direction should be more variable than the earlier implicit judgments, even if both types of judgment were based on the same representation. Furthermore, a constant error should be stronger for later explicit judgments than for earlier implicit ones; in the present study, the constant error was an offset in the counter-clockwise direction, which was observed in explicit judgments only. Thus, the assumption of a decaying memory representation of hand direction could account at least for two of the observed differences between explicit and implicit judgments. However, other differences observed are quite unlikely to result from decay characteristics of a single representation of hand direction. Which of the differences between implicit and explicit judgments of hand direction would disappear if the delays could be kept identical and which would remain is an important future question.

The opposite sequential effects for the explicit and the implicit judgments are perhaps the strongest evidence of different representations. They followed the pattern of an automatic (or implicit) facilitation of repetitions, and a controlled (or explicit) facilitation of alternations, as it has been observed in binary choice-reaction time tasks [52] and also in random-generation tasks [53]–[55]. In those tasks, there is only one type of response in each trial, a speeded response to an imperative signal or a self-determined choice from a given set of possible responses. Facilitation of repetitions tends to dominate at a fast rate of trials (e.g. inter-trial intervals of less than 1 s), but facilitation of alternations dominates at a slow rate.

In our task, there was only a single slow rate of trials, but there were two responses in each trial: the return movement for the implicit hand judgment and the subsequent slow movement for the explicit judgment. The two types of facilitation were observed in the two types of responses at a single slow rate. Facilitation of repetitions was seen in implicit judgments of hand direction, similar to previous observations on sequential effects in grip force production [56]–[57] and saccadic movements toward visual targets [58]. In contrast, for the explicit judgments, the automatic facilitation of repetitions was overridden by conscious expectations of the next type of judgment, which favor alternations rather than repetitions [52]–[55]. The co-occurrence of facilitation both of alternations and repetitions for explicit and implicit judgments, respectively, strongly suggests that they are based on distinct representations: the weight of the implicit representation is modulated by the past automatically, whereas the weight of the explicit representation is modulated by the expected future, in particular by subjective expectancies regarding the next type of judgment.

In addition, the facilitation of alternations was observable just with one prior trial history for the older adults in explicit judgments of both cursor and hand direction, whereas it required more than one prior trial history for the young adults. Thus, the facilitation of alternations becomes stronger with aging, suggesting that aging enhances controlled processes based on subjective expectations. A similar age-induced facilitation toward alternations was reported in a random generation task [59]. In contrast, there was no age difference in facilitation of repetitions in implicit judgments of hand direction. Again, these discrepant aging effects of sequence between the explicit and implicit judgments point to distinct representations that subserve the two types of judgment.

## Supporting Information

Text S1 **Appendix.**
(DOC)

## References

[1] Van Beers RJ, Sittig AC, Denier van der Gon JJ (1999) Integration of proprioceptive and visual position-information: An experimentally supported model. J Neurophysiol 81: 1355–1364. 10085361 10.1152/jn.1999.81.3.1355

[2] Cheng K, Shettleworth SJ, Huttenlocher J, Rieser JJ (2007) Bayesian integration of spatial information. Psychol Bull 133: 625–637. 17592958 10.1037/0033-2909.133.4.625

[3] Ernst MO, Bülthoff HH (2004) Merging the senses into a robust percept. Trends Cogn Sci 8: 162–169. 15050512 10.1016/j.tics.2004.02.002

[4] Ernst MO (2006) A Bayesian view on multimodal cue integration. In: Knoblich G, Thornton IM, Grosjean M., Shiffrar M, Editors. Human body perception from the inside out. Oxford: University Press. 105–131.

[5] Hay JC, Pick HL, Ikeda K (1965) Visual capture produced by prism spectacles. Psychon Sci 2: 215–216.

[6] Collins T, Schicke T, Röder B (2008) Action goal selection and motor planning can be dissociated by tool use. Cognition 109: 363–371. 19012884 10.1016/j.cognition.2008.10.001

[7] Reed CL, Betz R, Garza JP, Roberts RJ Jr (2010) Grab it! Biased attention in functional hand and tool space. Atten Percept Psychophys 72: 236–245. 20045892 10.3758/APP.72.1.236

[8] Müsseler J, Sutter C (2009) Perceiving one's own movements when using a tool. Conscious Cogn 18: 359–365. 19289291 10.1016/j.concog.2009.02.004

[9] Heuer H, Rapp K (2012) Adaptation to novel visuo-motor transformations: further evidence of functional haptic neglect. Exp Brain Res 218: 129–140. 22328066 10.1007/s00221-012-3013-z

[10] Bernier PM, Burle B, Vidal F, Hasbroucq T, Blouin J (2009) Direct evidence for cortical suppression of somatosensory afferents during visuomotor adaptation. Cereb Cortex 19: 2106–2113. 19126799 10.1093/cercor/bhn233

[11] Lajoie Y, Paillard J, Teasdale N, Bard C, Fleury M, et al (1992) Mirror drawing in a deafferented patient and normal subjects: visuoproprioceptive conflict. Neurology 42: 1104–1106. 1579235 10.1212/wnl.42.5.1104

[12] Balslev D, Christensen LOD, Lee J-H, Law I, Paulson OB, et al (2004) Enhanced accuracy in novel mirror drawing after repetitive transcranial magnetic stimulation-induced proprioceptive deafferentation. J Neurosci 24: 9698–9702. 15509758 10.1523/JNEUROSCI.1738-04.2004 PMC6730149

[13] Heuer H, Hegele M, Rand MK (2013) Age-related variations in the control of electronic tools. In: Schlick C, Frieling E, Wegge J, editors. Age-differentiated work systems. Heidelberg: Springer. 369–390.

[14] Heuer H, Sülzenbrück S (2012) Mind and movement. Psychol Res 76: 159–170. 21475957 10.1007/s00426-011-0332-9

[15] Heuer H, Sülzenbrück S (2013) Tool use in action. The mastery of complex visuomotor transformations. In: Prinz W, Beisert M, Herwig A, editors. Action science: Foundations of an emerging discipline. Cambridge: MIT Press. 37–62.

[16] Sperry RW (1950) Neural basis of the spontaneous optokinetic response produced by visual inversion. J Comp Physiol Psychol 43: 482–489. 14794830 10.1037/h0055479

[17] Von Holst E, Mittelstaedt H (1950) Das Reafferenzprinzip. Wechselwirkungen zwischen Zentralnervensystem und Peripherie. Naturwissenschaften 37: 464–476.

[18] Rand MK, Wang L, Müsseler J, Heuer H (2013) Vision and proprioception in action monitoring by young and older adults. Neurobiol Aging 34: 1864–1872. 23433708 10.1016/j.neurobiolaging.2013.01.021

[19] Bock O, Eckmiller R (1986) Goal-directed arm movements in absence of visual guidance: evidence for amplitude rather than position control. Exp Brain Res 62: 451–458. 3720877 10.1007/BF00236023

[20] Heuer H, Sangals J (1998) Task-dependent mixtures of coordinate systems in visuomotor transformations. Exp Brain Res 119: 224–236. 9535572 10.1007/s002210050336

[21] Heuer H, Sülzenbrück S (2012) The influence of the dynamic transformation of a sliding lever on aiming errors. Neuroscience 207: 137–147. 22309808 10.1016/j.neuroscience.2012.01.035

[22] Holmes NP, Crozier G, Spence C (2004) When mirrors lie: “Visual capture” of arm position impairs reaching performance. Cogn Affect Behav Neurosci 4: 193–200. 15460925 10.3758/cabn.4.2.193 PMC1314973

[23] Holmes NP, Spence C (2005) Visual bias of unseen hand position with a mirror: spatial and temporal factors. Exp Brain Res 166: 489–497. 16032401 10.1007/s00221-005-2389-4 PMC1343466

[24] Rossetti Y, Desmurget M, Prablanc C (1995) Vector coding of movement: Vision, proprioception, or both? J Neurophysiol 74: 457–463. 7472347 10.1152/jn.1995.74.1.457

[25] Heuer H, Hegele M (2008) Adaptation to visuo-motor rotations in younger and older adults. Psychol. Aging 23: 190–202. 10.1037/0882-7974.23.1.190 18361666

[26] Tewes U (1991) Hamburg-Wechsler-Intelligenztest für Erwachsene. (Rev ed). Toronto: Huber. 121 p.

[27] Neggers SFW, Bekkering H (2000) Ocular gaze is anchored to the target of an ongoing pointing movement. J Neurophysiol 83: 639–651. 10669480 10.1152/jn.2000.83.2.639

[28] Rand MK, Stelmach GE (2011) Effects of hand termination and accuracy requirements on eye-hand coordination in older adults. Behav Brain Res 219: 39–46. 21163306 10.1016/j.bbr.2010.12.008 PMC3062752

[29] Plooy A, Tresilian JR, Mon-Williams M, Wann JP (1998) The contribution of vision and proprioception to judgments of finger proximity. Exp Brain Res 118: 415–420. 9497148 10.1007/s002210050295

[30] Van Beers RJ, Wolpert DM, Haggard P (2002) When feeling is more important than seeing in sensorimotor adaptation. Curr Biol 12: 834–837. 12015120 10.1016/s0960-9822(02)00836-9

[31] Wang L, Sutter C, Müsseler J, Dangel RJZ, Disselhorst-Klug C (2012) Perceiving one's own limb movements with conflicting sensory feedback: the role of mode of movement control and age. Front Psychol 3: 289. 22908005 10.3389/fpsyg.2012.00289 PMC3414862

[32] Helbig HB, Ernst MO (2007) Knowledge about a common source can promote visual-haptic integration. Perception 36: 1523–1533. 18265835 10.1068/p5851

[33] Helbig HB, Ernst MO (2008) Visual-haptic cue weighting is independent of modality-specific attention. J Vis 8:21, 1–16. 10.1167/8.1.21 18318624

[34] Haaland KY, Harrington DL, Grice JW (1993) Effects of aging on planning and implementing arm movements. Psychol Aging 8: 617–632. 8292290 10.1037//0882-7974.8.4.617

[35] Seidler-Dobrin RD, Stelmach GE (1998) Persistence in visual feedback control by the elderly. Exp Brain Res 119: 467–474. 9588781 10.1007/s002210050362

[36] Simoneau M, Teasdale N, Bourdin C, Bard C, Fleury M, et al (1999) Aging and postural control: postural perturbations caused by changing the visual anchor. J Am Geriatr Soc 47: 235–240. 9988297 10.1111/j.1532-5415.1999.tb04584.x

[37] Goble DJ, Coxon JP, Wenderoth N, Van Impe A, Swinnen SP (2009) Proprioceptive sensibility in the elderly: Degeneration, functional consequences and plastic-adaptive processes. Neurosci Biobehav Rev 33: 271–278. 18793668 10.1016/j.neubiorev.2008.08.012

[38] Boisgontier MP, Olivier I, Chenu O, Nougier V (2012) Presbypropria: the effects of physiological ageing on proprioceptive control. Age 34: 1179–1194. 21850402 10.1007/s11357-011-9300-y PMC3448996

[39] Li SC, Sikström S (2002) Integrative neurocomputational perspectives on cognitive aging, neuromodulation, and representation. Neurosci Biobehav Rev 26: 795–808. 12470691 10.1016/s0149-7634(02)00066-0

[40] Welford AT (1981) Signal, noise, performance, and age. Hum Factors 23: 97–109. 7228049 10.1177/001872088102300109

[41] Bridgeman B, Lewis S, Heit G, Nagle M (1979) Relation between cognitive and motor-oriented systems of visual position perception. J Exp Psychol Hum Percept Perform 5: 692–700. 528967 10.1037//0096-1523.5.4.692

[42] Milner AD, Goodale MA (1995) The visual brain in action. Oxford: Oxford University Press. 248 p.

[43] Cohen YE, Andersen RA (2002) A common reference frame for movement plans in the posterior parietal cortex. Nat Rev Neurosci 3: 553–562. 12094211 10.1038/nrn873

[44] Desmurget M, Epstein CM, Turner RS, Prablanc C, Alexander GE, et al (1999) Role of posterior parietal cortex in updating reaching movements to a visual target. Nat Neurosci 2: 563–567. 10448222 10.1038/9219

[45] Medendorp WP, Goltz HC, Crawford JD, Vilis T (2005) Integration of target and effector information in human posterior parietal cortex for the planning of action. J Neurophysiol 93: 954–962. 15356184 10.1152/jn.00725.2004

[46] Georgopouplos AP, Schwartz AB, Kettner RE (1986) euronal population coding of movement direction. Science 233: 1416–1419. 3749885 10.1126/science.3749885

[47] Graziano MSA (1999) Where is my arm? The relative role of vision and proprioception in the neuronal representation of limb position. Proc Natl Acad Sci U S A 96: 10418–10421. 10468623 10.1073/pnas.96.18.10418 PMC17903

[48] Franz VH, Gegenfurtner K (2008) Grasping visual illusions: consistent data and no dissociation. Cogn Neuropsychol 25: 920–950. 10.1080/02643290701862449 18629739

[49] Smeets JBJ, Brenner E (2006) 10 years of illusions. J Exp Psychol Hum Percept Perform 32: 1501–1504. 17154790 10.1037/0096-1523.32.6.1501

[50] Shanks DR (2005) Implicit learning. In: Lamberts K, Goldstone R, editors. Handbook of Cognition London: Sage. 202–220.

[51] Paillard J, Brouchon M (1968) Active and passive movements in the calibration of position sense. In: Freedman SJ, editor. The Neuropsychology of spatially oriented behavior. Homewoood: Dorsey Press. 37–55.

[52] Kirby N (1980) Sequential effects in choice reaction time. In: Welford AT, editor. Reaction times. New York: Academic Press. 129–172.

[53] Falk R, Konold C (1997) Making sense of randomness: implicit encoding as a basis of judgment. Psychol Rev 104: 301–318.

[54] Heuer H, Kohlisch O, Klein W (2005) The effects of total sleep deprivation on the generation of random sequences of key presses, numbers, and nouns. Q J Exp Psychol 58A: 275–307. 10.1080/02724980343000855 15903118

[55] Mittenecker E (1953) Perseveration und Persönlichkeit. I. Teil: Experimentelle Untersuchungen. Z Exp Angew Psychol 1: 5–31.

[56] Johansson RS, Westling G (1988) Coordinated isometric muscle commands adequately and erroneously programmed for the weight during lifting task with precision grip. Exp Brain Res 71: 59–71. 3416958 10.1007/BF00247522

[57] Lukos JR, Choi JY, Santello M (2013) Grasping uncertainty: Effects of sensorimotor memories on high-level planning of dexterous manipulation. J Neurphysiol 109: 2937–2946. 10.1152/jn.00060.2013 PMC3680819 23554435

[58] Fecteau JH, Munoz DP (2003) Exploring the consequences of the previous trial. Nat Rev Neurosci 4: 435–443. 12778116 10.1038/nrn1114

[59] Heuer H, Janczyk M, Kunde W (2010) Random noun generation in younger and older adults. Q J Exp Psychol 63: 465–478. 10.1080/17470210902974138 19557669

