# Supplementary material for: Implicit and Explicit Representations of Hand Position in Tool Use
Source: PLoS One. 2013 Jul 19;8(7):e68471. doi: 10.1371/journal.pone.0068471 (PMC3716878; doi:10.1371/journal.pone.0068471)
Supplement: Text S1 — Appendix. (DOC) [file pone.0068471.s001.doc]

**Appendix**

Consider a proprioceptively and visually sensed characteristic such as the direction of one object such as the hand or two different objects such as the hand and a cursor in tool use, *P* and *V*. Optimal information integration is generally expressed as a weighted average [3]. We maintain this principle, but assume no perceptual fusion, but only biased proprioceptively and visually sensed directions *P*’ and *V*’:

*P*’ = *P* + w_v_ (*V*-*P*) = (1-w_v_) *P* + w_v_ *V*

*V*’ = *V* + w_p_ (*P*-*V*) = (1-w_p_) *V* + w_p_ *P*

with expected values

E(*P*’) = E(*P*) + w_v_ [E(*V*)-E(*P*)]

E(*V*’) = E( *V*) + w_p_ [E(*P*)-E(*V*)].

In these expressions, w_v_ is the weight of the visually sensed direction and w_p_ is the weight of the proprioceptively sensed direction. The larger the weight w_v_, the stronger is the visual bias of the proprioceptively sensed direction, and the larger the weight w_p_, the stronger is the proprioceptive bias of the visually sensed direction.

The variances of the biased directions, assuming stochastic independence of P and V, are:

var(*P*’) = (1- w_v_)^2^ var(*P*) + w_v_^2^ var(*V*)

var(*V*’) = (1- w_p_)^2^ var(*V*) + w_p_^2^ var(*P*).

In Figure 1, the relative variances are plotted as functions of the biases (or weights) w_v_ and w_p_ for different ratios of var(*V*) and var(*P*):

var(*P*’) / var(*P*) = (1- w_v_)^2^ + w_v_^2^ var(*V*) / var(*P*)

var(*V*’) / var(*V*) = (1- w_p_)^2^ + w_p_^2^ var(*P*) / var(*V*).

For perfect integration, the variances of the biased signals reach minima at w_v_ = var(*P*) / [var(*P*) + var(*V*)] and at w_p_ = var(*V*) / [var(*P*) + var(*V*)], w_v_ + w_p_ =1. This corresponds to the Maximum Likelihood Estimation model of information integration. In the case of coupling, w_v_+w_p_<1. With appropriate weights, the variances of the biased sensory signals are reduced, but fall short of reaching the minimum.
